# Editorial: The African food environments

**DOI:** 10.3389/fpubh.2023.1181096

**Published:** 2023-06-16

**Authors:** Amos Laar, Kaleab Baye, Francis Zotor, Gershim Asiki, Anna Lartey

**Affiliations:** ^1^Department of Population, Family and Reproductive Health, School of Public Health, University of Ghana, Accra, Ghana; ^2^Research Center for Inclusive Development in Africa (RIDA), Nutrition and Food Systems Division, Addis Ababa, Ethiopia; ^3^Center for Food Science and Nutrition, Addis Ababa University, Addis Ababa, Ethiopia; ^4^Fred Binka School of Public Health, University of Health and Allied Sciences, Hohoe, Ghana; ^5^African Population and Health Research Center, Nairobi, Kenya; ^6^Department of Nutrition and Food Science, University of Ghana, Accra, Ghana

**Keywords:** Africa, food environments, nutrition transition, non-communicable diseases, public health

In many respects, the continent of Africa is undergoing multiple transitions of which the nutrition transition is the most prominent—from a public health perspective. Popkin et al. (1) define nutrition transition as shifts in diets at the population level coinciding with globalization and changes in a country's overall development, food environments, and food systems. One consequence of the nutrition transition is the increase in nutrition-related non-communicable diseases (NCDs) such as obesity, type 2 diabetes, cardiovascular diseases, and certain cancers. Although NCDs are a global public health problem, their rate of increase in low and middle income countries (LMICs) is staggering (2). The surge has been linked to modifiable environmental factors, including physical and social environments—in which people live, work, and eat. Referred to as the food environment—the physical and metaphorical interface that mediates people's food acquisition and consumption within the wider food system (3), available evidence show that it is a key determinant of population health. Unhealthy food environments avail unhealthy foods, and drive unhealthy diets—facilitating the excessive consumption of ultra-processed energy-dense nutrient-poor foods, rather than healthier alternatives such as unrefined cereals, seeds, nuts, fruits, and vegetables.

While the papers included in this Research Topic do not cover the current COVID-19 pandemic, the Russia-Ukraine war, and other global crises such as climate change, the impact of these crises on the food environments, food availability, food prices, food affordability, and food security is real (4, 5). The pandemic and related global economic recession are severe setbacks to already insufficient progress toward meeting the global nutrition targets set for 2025 for stunting, wasting, maternal anemia and breastfeeding (6), and now threaten to exacerbate maternal and child undernutrition across low LMICs (7). Africa is particularly hard hit, having been disproportionally affected by the combined effects of other conflicts, and climate change.

However, our current understanding of the dynamics of the rapidly transitioning African food environments is limited. While hunger and food insecurity still persist, other forms of malnutrition such as obesity and related NCDs have emerged. Toward addressing malnutrition in all its forms, improving food environments in Africa is an urgent priority. Data-driven approaches, fit-for-local policies and actions and responding to all forms of malnutrition are needed. Fit-for-local purpose data can give insights into which policies may be more effective in combating the multiple forms of malnutrition in Africa. This Research Topic on African Food Environments aimed to solicit practice-impacting and policy-influencing evidence from researchers and practitioners working on the African food environments. It comprises a collection of nine papers from six African countries—eight empiric studies, and a review.

Two studies from South Africa characterized the operations of the street food enterprises (Mahopo et al.) and examined the nexus between food security indicators and anthropometric health. The authors identified opportunities for improving the food environments of a rural South African setting through the implementation of government policies that target street vendors. The authors recommended that, government in partnership with non-state actors deliver such interventions as training and microfinance to improve the business skills of street food vendors while promoting food safety and nutritious foods. Harper et al. estimated the prevalence of double burden of malnutrition in select South African households, and showed that about 70.2% of all stunted children lived with an overweight or obese adult.

In Addis Ababa, Ethiopia, Trübswasser et al. assessed factors influencing adolescents' dietary behaviors in the school and home environments. The authors reported pervasive advertising and availability of unhealthy ultra-processed foods and beverages within the 0.5 km radius around the schools. In their interpretations of the association of unhealthy food environments and outcomes like dietary diversity and nutritional status, the authors acknowledged that their study was not appropriately powered and that the cross-sectional design did not allow for control of temporal factors. Nevertheless, the study provided unique characterization of the food environment of adolescents in Addis Ababa, leading to the conclusion that unhealthy food environments as observed in the study could be an impediment to the success of interventions that promote healthy dietary behaviors.

Tione et al. examined the role of farm input subsidies in wasting prevention among Malawian children under-5 years. Data from the study suggest that input subsidies can speed up wasting reduction among children under-5 years through pathways such as increased maize production, sustained food availability. In Uganda, Nankumbi et al. assessed vitamin A-rich food consumption and its predictors among women of reproductive age from an orange-fleshed sweet potato-growing households of Uganda. This work showed that knowledge about vitamin A did not predict vitamin A rich food consumption.

Three studies from Ghana explored different dimensions of the school food environment. Amevinya et al. examined the extent and nature of food and beverage advertising around primary and junior high schools in Ghana's most populous and urbanized region, Greater Accra. As in the Ethiopian study, Amevinya et al. report a pervasive advertising of sugar-sweetened and alcoholic beverages in the studied schools. They recommend policy action such as restriction of marketing of unhealthy foods, and zoning regulation to limit the exposure to children of unhealthy food advertisements. A related study by  Adjei et al. reveals widespread availability of ultra-processed foods in modern retail outlets in the same setting. Toward a healthier food retail environment, public health, and food regulators, in partnership with other stakeholders need to institute measures that improve availability of healthy foods within supermarkets and mini-marts. Nanema et al. explored Accra-based food retailers' perceptions and appreciation of “healthiness of food” as a concept. They also documented measures that food retailers adopt to encourage healthy food choices. Accra-based retailers have a fair understanding of what constitutes healthy food–exhibiting limited knowledge of the connection between very salty, very sugary, and very fatty foods and health outcomes, the researchers reported. Retailers in Accra would benefit from interventions that improve their food, health, and nutrition literacy.

Presenting regional data (focusing on School Meal Programs in Africa) from the 2019 Global Survey of School Meal Programs, Wineman et al. commended the “home-grown school feeding philosophy” in Africa. These favor national ownership and domestic food procurement, with positive externalities spanning social, and economic dimensions. A salient, but rather worrying finding from the survey was the limited attention given to overweight/obesity in school meal programs in Africa (Wineman et al.). Only about 10% of the School Meal Programs identified overweight/obesity prevention as an objective. As Africa becomes increasingly urbanized, with transitioning dietary practices that favor processed foods, high in salt, high in sugar, high in unhealthy fat foods with limited consumption of fruits and vegetables but (8), policy and program inattention to the multiple burdens of malnutrition in the continent is concerning. Facing a syndemic of undernutrition, overweight/obesity and other diet-related NCDs, it is crucial that population health policies are responsive to all of these realities. Complementary food environment policies and practices that respond to malnutrition in all its forms—a mix of both low and high agency policy interventions—interventions that seek to “inform and empower”; those that “guide and influence” and those that “incentivize (consumption of healthier foods),” and dis-incentivize/discourage intake of unhealthy foods are urgently needed (9).

## Data availability statement

The original contributions presented in the study are included in the article/supplementary material, further inquiries can be directed to the corresponding author.

## Author contributions

ALaa: conceptualization and writing of first draft. KB, FZ, GA, and ALar: reviewing and editing of first and final drafts. All authors contributed to the article and approved the submitted version.
